# Comparison of Brain Activation Patterns during Olfactory Stimuli between Recovered COVID-19 Patients and Healthy Controls: A Functional Near-Infrared Spectroscopy (fNIRS) Study

**DOI:** 10.3390/brainsci11080968

**Published:** 2021-07-23

**Authors:** Roger C. Ho, Vijay K. Sharma, Benjamin Y. Q. Tan, Alison Y. Y. Ng, Yit-Shiang Lui, Syeda Fabeha Husain, Cyrus S. Ho, Bach X. Tran, Quang-Hai Pham, Roger S. McIntyre, Amanda C. Y. Chan

**Affiliations:** 1 Department of Psychological Medicine, Yong Loo Lin School of Medicine, National University of Singapore, Singapore 119228, Singapore; pcmrhcm@nus.edu.sg (R.C.H.); pcmhsh@nus.edu.sg (C.S.H.); 2 Institute of Health Innovation and Technology (iHealthtech), National University of Singapore, Singapore 117599, Singapore; 3 Department of Medicine, Yong Loo Lin School of Medicine, National University of Singapore, Singapore 119228, Singapore; vijay_kumar_sharma@nuhs.edu.sg; 4 Department of Medicine, National University Hospital, Singapore 119228, Singapore; benjaminyqtan@gmail.com (B.Y.Q.T.); alison_yy_ng@nuhs.edu.sg (A.Y.Y.N.); amanda_chan@nuhs.edu.sg (A.C.Y.C.); 5 Department of Psychological Medicine, National University Health System, Singapore 119228, Singapore; yit_shiang_lui@nuhs.edu.sg; 6 Bloomberg School of Public Health, Johns Hopkins University, Baltimore, MD 21205, USA; bach@jhu.edu; 7 Institute for Preventive Medicine and Public Health, Hanoi Medical University, Hanoi 116001, Vietnam; 8 Institute for Global Health Innovations, Duy Tan University, Da Nang 550000, Vietnam; phamquanghai@duytan.edu.vn; 9 Faculty of Medicine, Duy Tan University, Da Nang 550000, Vietnam; 10 Mood Disorders Psychopharmacology Unit, University Health Network, University of Toronto, Toronto, ON M5G 2C4, Canada; roger.mcintyre@uhn.ca; 11 Department of Psychiatry, University of Toronto, Toronto, ON M5T 1R8, Canada; 12 Canadian Rapid Treatment Center of Excellence, Mississauga, ON L5C 4E7, Canada

**Keywords:** COVID-19, functional near-infrared spectroscopy (fNIRS), olfactory stimuli, sniffin’ sticks 12-identification test

## Abstract

Impaired sense of smell occurs in a fraction of patients with COVID-19 infection, but its effect on cerebral activity is unknown. Thus, this case report investigated the effect of COVID-19 infection on frontotemporal cortex activity during olfactory stimuli. In this preliminary study, patients who recovered from COVID-19 infection (*n* = 6) and healthy controls who never contracted COVID-19 (*n* = 6) were recruited. Relative changes in frontotemporal cortex oxy-hemoglobin during olfactory stimuli was acquired using functional near-infrared spectroscopy (fNIRS). The area under curve (AUC) of oxy-hemoglobin for the time interval 5 s before and 15 s after olfactory stimuli was derived. In addition, olfactory function was assessed using the Sniffin’ Sticks 12-identification test (SIT-12). Patients had lower SIT-12 scores than healthy controls (*p* = 0.026), but there were no differences in oxy-hemoglobin AUC between healthy controls and patients (*p* > 0.05). This suggests that past COVID-19 infection may not affect frontotemporal cortex function, and these preliminary results need to be verified in larger samples.

## 1. Introduction

Globally, as of May 6, 2021, there have been 154,640,649 confirmed cases of Coronavirus disease-2019 (COVID-19), including 3,232,285 deaths, reported to the World Health Organization (WHO) [1]. Since the outbreak of the COVID-19 pandemic, medical professionals have a better understanding of the pathogenesis and clinical features of COVID-19 infection. COVID-19 creates severe respiratory distress and other systemic complications involving multiple organ systems, due to a cascade of immunological responses [2]. Patients who suffered from COVID-19 infection may develop neurological complications, including acute myelitis, cerebrovascular accidents, cerebral venous thrombosis, Guillain-Barré syndrome, meningoencephalitis, posterior reversible encephalopathy syndrome and seizures [3]. Common neuropsychiatric symptoms of COVID-19 infection include change in mental state, delirium, giddiness, gustatory impairment, myalgia, headache [3] and cognitive impairment [4]. In addition to the above symptoms, COVID-19 infection is known to cause olfactory dysfunction [5]. Recent studies have focused on developing questionnaires and a smell identification test that assess olfactory dysfunction [6,7]. It has been reported that the prevalence of olfactory dysfunction is 73.1%, with a male to female ratio of 1:3 [8]. It was also reported that complete recovery of olfactory dysfunction occurs in 31.8% of COVID-19 survivors [8].

Olfactory dysfunction may be due to olfactory cleft opacification and olfactory bulb degeneration, detected by magnetic resonance imaging (MRI) of the olfactory bulb and computer tomography of the paranasal sinus [9]. Furthermore, there has been a report on the association between opacification in the olfactory cleft and the degree of loss of smell [10]. Olfactory dysfunction is thought to manifest when viral replication in the non-neural olfactory cells indirectly cause damage to the olfactory receptor nerves [11]. The COVID-19 virus binds to the human angiotensin converting enzyme ACE2 receptor, and it may thus target other cells that express this protein, such as neurons in the brain [5]. Viruses have been shown to be transported along synapses connecting the peripheral olfactory epithelium and the central nervous system. The first target regions are part of the olfactory system, including the olfactory bulb and amygdala. The virus may then spread to other tissues and induce neurodegenerative symptoms such as epilepsy, motor and cognitive symptoms [5]. In addition, recent studies have linked the incidence of acute respiratory failure with COVID-19 infection of the brainstem. It has been postulated that the COVID-19 virus causes dysfunction of the respiratory center by spreading from the olfactory bulb to the olfactory nerves, the rhinencephalon and, finally, the brainstem [12].

Despite the potential neuropathogenesis, neuroimaging studies on patients with COVID-19 and other respiratory infections are limited. Electroencephalography of patients with COVID-19 infection have shown frontal lobe abnormalities [13,14], while positron emission tomography (PET) of these patients has identified altered glucose metabolism in several brain areas. These include hypometabolism in the bilateral parahippocampal and fusiform gyri, left insula [15], dorsolateral prefrontal cortex, bilateral frontal eye fields and right anterior cingulate cortex, as well as hypermetabolism in the left orbitofrontal cortex, right posterior parietal cortex and right thalamus [16]. Grey matter volume loss detected by MRI occurred in the right orbitofrontal cortex of patients with post-infectious olfactory loss [17], decreased functional connectivity of the chemosensory network occurred in patients with chronic peripheral smell loss [17] and hypometabolism of the medial and lateral temporal cortex occurred in patients with olfactory dysfunction after an upper respiratory tract infection [18]. Taken together, the literature suggests that COVID-19 infection may affect brain function and that viral-associated loss of smell may be linked to long-term changes in the brain. Still, there is a gap in the literature about the neurophysiological response to direct olfactory stimulation in patients with viral-associated smell loss. Hence, neurobiological investigations with technologies that allow neuroimaging data to be acquired during direct olfactory stimulation, such as functional near-infrared spectroscopy (fNIRS), are needed.

fNIRS enables real-time monitoring of hemodynamic changes in the cerebral cortex, because light in the near-infrared spectrum has the unique property of passing though tissues and being preferentially absorbed by hemoglobin in the cerebral cortex [19]. The absorbance spectra of hemoglobin is dependent on its binding with oxygen, which enables fNIRS devices to continuously detect relative changes in both oxy-hemoglobin and deoxy-hemoglobin in the cortical regions being studied [20]. According to a phenomenon called neurovascular coupling, fNIRS signals are believed to be a surrogate measure of the underlying neural activity [21]. Regional neuron activity triggers an increase in blood flow and volume that is many times higher than the metabolic demand. Therefore, cerebral hemodynamic response typically involves a large increase in oxy-hemoglobin and a simultaneous slight decrease in deoxy-hemoglobin [22]. Since there is a net change in oxy-hemoglobin, oxy-hemoglobin is used as a marker of cerebral activity [23].

Although near-infrared light cannot reach brain areas below the cortex, fNIRS has several practical advantages in both research and clinical settings compared to conventional neuroimaging modalities, such as functional magnetic resonance imaging (fMRI). Both fMRI and fNIRS are non-invasive and non-ionizing modalities that map brain activity by measuring changes in haemoglobin concentration. Studies that have used fNIRS and fMRI concurrently to measure activity during mental tasks have found that fNIRS signals are highly correlated with fMRI blood oxygen level-dependent (BOLD) signals recorded in the same neuroanatomical regions [24]. fNIRS offers an alternative to fMRI for olfactory research, because it is economical, quiet, does not require restraints, tolerant to motion, does not exclude special populations (such as individuals with claustrophobia or metal implants) [19] and does not require special equipment to deliver odorants [25]. Importantly, abnormal fNIRS signals during various olfactory stimuli have been reported in the frontal, temporal and/or parietal cortex of patients with chronic sinusitis [26], multiple chemical sensitivity [27], attention-deficit/hyperactivity disorder [28], subjective memory complaints, mild cognitive impairment and very mild Alzheimer’s disease [29], relative to healthy controls.

To the best of our knowledge, no research article has been published on fNIRS of individuals with COVID-19 infection since the outbreak of the pandemic. Such an instigation may identify abnormalities in olfactory and cerebral cortex function induced by the COVID-19 virus. Due to the lockdown and other social measures, we were not able to carry out large scale recruitment or recruit patients while they were infected with COVID-19. Thus, we compared fNIRS signals during olfactory stimuli between individuals with and without a history of COVID-19 infection in this case report. Among the regions of the brain associated with olfactory processing, the role of the orbitofrontal cortex is the most well established [25]. Hence, we hypothesized that oxy-hemoglobin changes during olfactory stimuli in the frontotemporal cortex would be lower in patients who recovered from COVID-19 infection than healthy controls.

## 2. Materials and Methods

### 2.1. Participants

This pilot case report included six healthy controls and six patients who had recovered from COVID-19 infection. All participants were at least 21-years old. Healthy controls were recruited from the community, while patients were recruited from and managed at our tertiary institution, the National University Hospital, Singapore, between 1 April to 31 October 2020. COVID-19 patients were contacted after they were discharged from the hospital and posed no significant risk of infecting others, including the researchers. The inclusion criterion for patients was a confirmed diagnosis of COVID-19 infection and a positive real-time reverse-transcription polymerase chain reaction assay of throat swab samples. Healthy controls were included if they had never contracted COVID-19 and had a negative COVID-19 swab test. The exclusion criteria for all participants were a history of neurological illnesses; traumatic brain injury; psychiatric disorder; intellectual disability; active cerebrovascular, respiratory, hepatic, renal disease, malignancy, chronic nasal conditions and previous nasal surgery. In addition, participants with rhinitis, history of sinunasal disease or known hyposmia for reasons other than past COVID-19 infection were excluded.

This study complied with the ethical standards of the Declaration of Helsinki and the ethical principles in the Belmont Report. It was approved by the Domain Specific Review Board (DSRB) of the National Healthcare Group, Singapore (protocol number 2020/00832). Written informed consent was obtained from participants prior to the commencement of the study.

### 2.2. Olfactory Assessment and Assessment of Other Symptoms

All participants refrained from smoking, eating and drinking 30 min prior to participating in this study. Olfactory function was assessed with the Sniffin’ Sticks 12-identification test (SIT-12; MediSense, Groningen, The Netherlands) [30]. The SIT-12 is a psychophysical, semi-objective assessment of olfactory performance [31] that has been validated in previous studies involving COVID-19 patients [32]. The test consists of 12 Sniffin’ sticks that resemble the 12 different odorants, including peppermint, fish, coffee, banana, orange, rose, lemon, pineapple, cinnamon, cloves, leather and licorice. Each Sniffin’ stick resembles a felt-tip pen containing a tampon and a liquid odorant dissolved in propylene glycol (4% dilution with a total volume of 4 mL) [28]. Sniffin’ Sticks were sequentially placed 2–3 cm in front of the subject’s nose for 3 s, and participants needed to choose one item out of four items on a card that was most closely associated with the scent [33]. The total number of correctly identified odorants was recorded as the SIT-12 score [33]. Scores from 0–6, 7–10 and 11–12 indicate anosmia, hyposmia and normosmia, respectively [34].

### 2.3. fNIRS Measurements

A 52-channel fNIRS system (ETG-4000. Hitachi Medical Co., Tokyo, Japan) was used in this study. This fNIRS device used two near-infrared light wavelengths (695 and 830 nm) to measure relative oxy-hemoglobin and deoxy-hemoglobin changes at a sampling rate of 10 Hz. Emitter and detector optodes were placed 3 cm apart. The cortical areas between each emitter and detector pairs are known as a channel. Anatomically, channels correspond to cortical regions 2–3 cm beneath the skin and scalp surface [35]. Optodes were placed on the forehead and scalp, with the lowest optodes placed along the T4-Fpz-T3 line, defined by the 10/20 system. This arrangement allowed for hemoglobin changes in the bilateral prefrontal cortex, frontopolar cortex and the anterior regions of the superior and middle temporal cortices to be measured. These approximate channel locations were based on the anatomical craniocerebral correction of the international 10/20 system.

### 2.4. Olfactory Stimuli

Olfactory stimuli during fNIRS measurements began with a 30-s rest period, followed by four 10-s olfactory stimulus periods that alternated with four 50-s rest periods [28]. A different odorant was presented for each stimulus period using Sniffin’ Sticks (Burghart Instruments, Germany) that were held 1 cm in front of the participant’s nostrils. The odorants were apple, garlic, chocolate and sesame oil [36], and these were presented in this order. Participants were asked to keep their eyes closed, keep their head and body still and breathe naturally throughout the fNIRS measurement.

### 2.5. fNIRS Signal Processing

**Figure 1:**
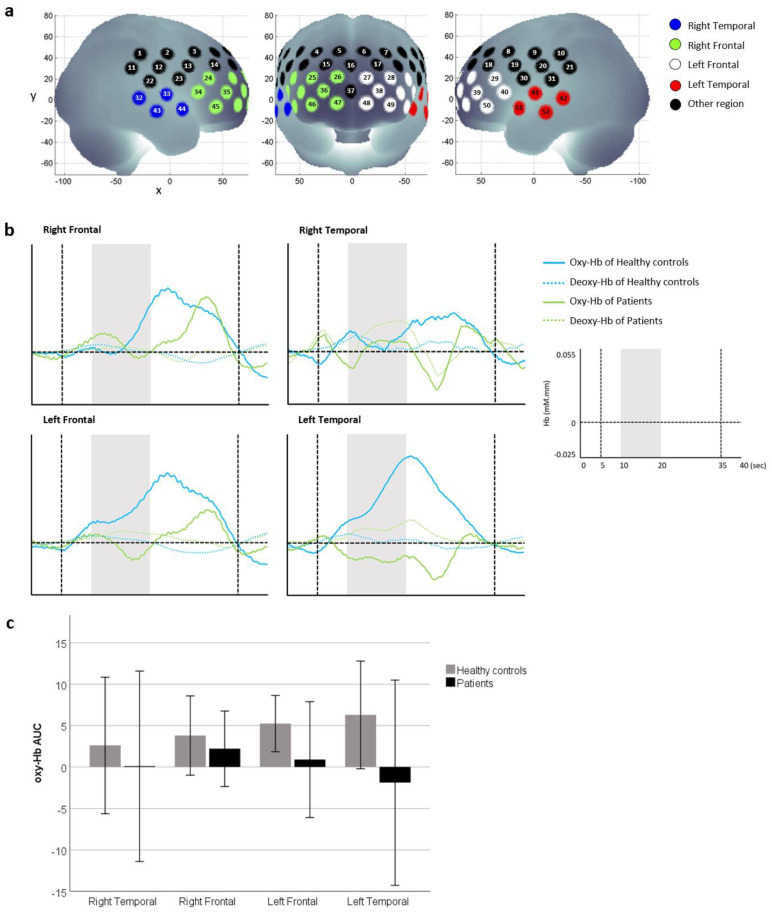
(**a**) Four regions called the right and left frontal and temporal regions were studied. (**b**) Average oxy-hemoglobin and deoxy-hemoglobin waveforms of patients who recovered from COVID-19 infection and healthy controls who never contracted COVID-19 were generated for each region. Grey areas indicate the olfactory stimulus period, and vertical dotted lines indicate the time period used to calculate the AUC of oxy-hemoglobin. (**c**) Comparison of oxy-hemoglobin AUC between healthy controls and patients. Data are presented as mean ± SD. There were no differences between groups when oxy-hemoglobin AUC at each region were compared using Mann–Whitney U tests (*p* > 0.05).

The fNIRS signals were processed according to the method described by Schecklmann et al. [28]. Oxy-hemoglobin, deoxy-hemoglobin and total hemoglobin were derived from optical densities using the modified Beer–Lambert law. A moving average factor of five was applied to remove short-term motion artefacts. Slow drifts were removed using linear fitting between a 10-s baseline at the end of the pre-stimulus rest period and a 10-s post-stimulus baseline period that begins 10 s into the post-stimulus rest period. Channels with body movement artefacts or high and low frequency noise were removed from further analysis. An average oxy-hemoglobin and deoxy-hemoglobin waveform of the four stimulus periods was generated for remaining channels. Since oxy-hemoglobin is typically used as a marker of cortical activity, the area under curve (AUC) of oxy-hemoglobin for the time interval 5 s before and 15 s after olfactory stimuli [28] was derived for each participant and region. The four regions of interest were the left and right frontal and temporal cortex (Figure 1a). The AUC of oxy-hemoglobin was used in further statistical analysis.

### 2.6. Statistical Analysis

The effect of diagnostic group (i.e., COVID-19 infection vs. healthy controls) on continuous and categorical variables was determined using Mann–Whitney U test and Pearson’s chi-squared test, respectively. Continuous variables were age, SIT-12 score and AUC of oxy-hemoglobin at each region of interest. Categorical variables were gender and handedness. All tests were two-tailed, with a significance of *p* ≤ 0.05. Statistical analysis was performed using SPSS (Version 26, Armonk, NY, USA: IBM Corp).

## 3. Results

### 3.1. Sample Characteristics

Healthy controls and patients who had recovered from COVID-19 infection did not differ in gender (*p* = 1.0) and handedness (*p* = 1.0) (see Table 1). Compared to healthy controls, patients were older but within the adult age range, and had lower SIT-12 scores (*p* = 0.002). The median time between recovery from COVID-19 infection to fNIRS measurement was 20.6 (range 16.6–27.6) weeks. Amongst healthy controls, five were normosmic (83.3%), and one was dysosmic (16.7%). Amongst patients, two were normosmic (33.3%), two were dysosmic (33.3%) and two were anosmic (33.3%).

### 3.2. Cortical Oxy-Haemoglobin during Olfactory Stimuli

Averaged oxy-hemoglobin waveforms (see Figure 1b) showed that relative oxy-hemoglobin changes during and after olfactory stimuli may have been higher in healthy controls than patients who recovered from COVID-19 infection, particularly in the left frontal and temporal region. However, there was no difference in oxy-hemoglobin AUC between healthy controls and patients in any region (see Figure 1c).

## 4. Discussion

Research on COVID-19-associated smell loss is important, because olfactory dysfunction has been linked to reduced mortality, quality of life, appetite and immunity. Loss of smell may also worsen medical illnesses and raises safety concerns, due to the inability to detect noxious environmental elements, such as fire, gas leaks and spoiled food [37]. This pilot case report found that patients who recovered from COVID-19 infection demonstrated lower olfactory function than healthy controls. Reduced SIT-12 scores in this sample of patients is consistent with previous studies that reported a persistent loss of smell even after patients with this symptom recovered from COVID-19 infection [38,39]. The cellular basis for these observations may be explained by a study describing the presence of virus transcripts and of SARS-CoV-2-infected cells in the olfactory mucosa of patients with long-term persistence of COVID-19-associated loss of smell [40].

There were no differences in oxy-hemoglobin AUC during olfactory stimuli between healthy controls and patients, suggesting that frontotemporal function is not affected by past COVID-19 infection. Still, several routes by which the brain may be infected have been proposed, including entry through the olfactory nerve, transsynaptic transfer across infected neurons, vascular endothelium infection and migration of leukocytes across the blood–brain barrier [41]. Furthermore, previous reports have demonstrated that the COVID-19 virus can infect cells and cause neuronal death in vitro. On the other hand, analysis of cerebral spinal fluid samples and post-mortem brain tissues has provided inconsistent evidence for the neuroinvasion of COVID-19 [42]. When reviewing findings from animal models, Butowt et al. [43] concluded that the evidence for the olfactory route to brain infection is weak, and that the loss of smell does not indicate that the virus has entered the brain. In short, the mechanisms behind the neurological symptoms in COVID-19 infection and the potential for neuroinvasion of the virus in humans is still not clear [44]. Given that the available data on how the COVID-19 virus interacts with the brain and its vasculature is limited, the basic question of whether neurological manifestations of COVID-19 reflects brain invasion remains unanswered [42]. Continued efforts to study the acute and long-term effects of COVID-19 infection on the brain, using various techniques, including neuroimaging, is essential to enhance our understanding of the neurogenesis of this virus. Thus, repeating the fNIRS measurements during olfactory stimuli in a larger sample of individuals with and without a history of COVID-19 infection may be relevant. Furthermore, olfactory training is currently the only evidence-based therapy available for patients who have not regained their sense of smell after one month since the onset of COVID-19 induced olfactory dysfunction [37]. Future neuroimaging research on the effect of olfactory training may be of interest as well.

There are several limitations in this preliminary fNIRS study. Firstly, a small sample of participants were recruited due to quarantine measures during the pandemic. Consequently, this case report did not have adequate power to demonstrate statistically significant differences in cortical oxy-hemoglobin AUC between healthy controls and patients who recovered from COVID-19 infection. Secondly, patients were older than healthy controls. Regardless, all participants were within the adult age range, and the median age of healthy controls and patients who recovered from COVID-19 was under 40 years. Interestingly, a recent study reported that young adults who recovered from COVID-19 infection were prone to develop olfactory dysfunction [45]. Hence, we believe the effect of older age on measured outcomes was minimal. Thirdly, participants in this preliminary study were not exposed to a control odor during fNIRS measurements. Therefore, the findings of increased oxy-hemoglobin during olfactory stimuli in healthy controls need to be verified in a larger sample of individuals who are exposed to both experiment and control odors. Fourthly, the SIT-12 is a screening tool that does not have the same diagnostic accuracy as the standard Sniffin’ Sticks test, which comprises of threshold, discrimination and identification subtests. Fifthly, not all healthy controls were normosmic, and a fraction of patients were normosmic. Lastly, the median time of the fNIRS scan and recovery of COVID-19 infection was 20.6 weeks and not immediate after COVID-19 infection. A recent study found that there was a time-dependent increase in smell scores in healthcare workers who had recovered from COVID-19 infection [46]. We could not recruit patients soon after they were discharged from the hospital due to safety measures and concerns over the spread of COVID-19 to the research team and healthy controls. Despite these limitations, this has been the first functional neuroimaging case report on patients with who recovered from COVID-19 infection.

## 5. Conclusions

A history of COVID-19 infection was found to be associated with lower olfactory function assessed by the SIT-12, but not frontotemporal cortex hemodynamic response to olfactory stimuli measured with fNIRS. Nevertheless, fNIRS investigations on a larger sample of individuals with and without a history of COVID-19 infection may provide additional objective evidence to support the persistence of olfactory dysfunction detected by semi-objective smelling tests and self-reported questionnaires.

## Figures and Tables

**Table 1:** Demographic and clinical variables.

	Healthy Controls (*n* = 6)	Patients Who Had Recovered from COVID-19 Infection (*n* = 6)	*p*-Value
Median age in years (range)	32 (30–33)	39.5 (36–48)	**0.002**
Gender			1.000
Male *n* (%)	4 (66.7%)	4 (66.7%)	
Female *n* (%)	2 (33.3%)	2 (33.3%)	
Handedness			1.000
Right *n* (%)	6 (100%)	5 (83.3%)	
Left *n* (%)	0	1 (16.7%)	
Median SIT-12 score (range)	11.5 (10–12)	7.5 (4–11)	**0.026**
Median duration in weeks between recovery from the COVID-19 infection and fNIRS measurement (range)		20.6 (16.6–27.6)	

*p* ≤ 0.05 are in bold.

## Data Availability

Data can be obtained from the corresponding author upon request.
